# Probable COVID-19 infection is associated with subsequent poorer mental health and greater loneliness in the UK COVID-19 Mental Health and Wellbeing study

**DOI:** 10.1038/s41598-022-24240-3

**Published:** 2022-12-02

**Authors:** Sarah Wilding, Daryl B. O’Connor, Eamonn Ferguson, Seonaid Cleare, Karen Wetherall, Ronan E. O’Carroll, Kathryn A. Robb, Rory C. O’Connor

**Affiliations:** 1 School of Psychology, University of Leeds, Leeds, UK; 2 School of Psychology, University of Nottingham, Nottingham, UK; 3 Suicidal Behaviour Research Laboratory, Institute of Health and Wellbeing, University of Glasgow, Glasgow, Scotland; 4 Division of Psychology, University of Stirling, Stirling, Scotland; 5 Institute of Health and Wellbeing, University of Glasgow, Glasgow, Scotland

**Keywords:** Psychology, Human behaviour

## Abstract

The COVID-19 pandemic has been associated with psychological distress. In addition to physical effects including fatigue and cognitive impairment, contracting COVID-19 itself may also be related to subsequent negative mental health outcomes. The present study reports data from a longitudinal, national survey of the UK adult population investigating whether contracting suspected or confirmed COVID-19 at the early stages of the pandemic (March–May 2020) was associated with poorer mental health outcomes in May/June 2020, October/November 2020 and June/July 2021. A quota survey design and a sampling frame that permitted recruitment of a national sample (n = 3077) were utilised. Experience of contracting COVID-19 during the first UK lockdown was assessed along with levels of depression, anxiety, mental wellbeing and loneliness. Around 9% of participants reported contracting COVID-19 in March/May 2020 (waves 1–3) with just under 13% of the overall sample reporting COVID-19 at any one of the first three time points. Compared to those without probable COVID-19 infection, participants with probable COVID-19 had poorer mental health outcomes at follow-up with these effects lasting up to 13 months (e.g., May/June 2020:OR_depression_ = 1.70, *p* < 0.001; OR_anxiety_ = 1.61, *p* = 0.002; Oct/Nov 2020, OR_depression_ = 1.82, *p* < 0.001; OR_anxiety_ 1.56, *p* = 0.013; June/July 2021, OR_depression_ = 2.01, *p* < 0.001; OR_anxiety_ = 1.67, *p* = 0.008). Having a pre-existing mental health condition was also associated with greater odds of having probable COVID-19 during the study (OR = 1.31, *p* = 0.016). The current study demonstrates that contracting probable COVID-19 at the early stage of the pandemic was related to long-lasting associations with mental health and the relationship between mental health status and probable COVID-19 is bidirectional.

## Introduction

The COVID-19 pandemic was associated with psychological distress particularly during the initial lockdown in April 2020^1,2^, and the relationship between COVID-19 and mental health has been demonstrated worldwide^3^. The negative association between the pandemic and mental health is likely due to a variety of reasons, including the negative impact of lockdowns, physical distancing, restricted movement, quarantine, high levels of isolation, insecure employment, and childcare issues^4^. A key study, using a large dataset in the United States (US), found that that the increases in psychological distress observed since the emergence of COVID-19 were explained by a range of pandemic-related stressors including perceived infection risk and risk of death, financial concerns and enforced isolation^5^. Mental health outcomes associated with the COVID-19 pandemic have also been shown to be poorer in specific groups of people including women, younger individuals and those with pre-existing mental and physical health conditions^6,7^. However, a recent meta-analysis comparing mental health before versus during the pandemic did not find evidence of more pronounced effects on mental health symptoms in individuals with a pre-existing mental health conditions^2^. Nevertheless, early findings from the current study, known as the UK COVID-19 Mental Health and Wellbeing study, found that individuals with a pre-existing mental health condition reported poor mental health early in the UK lockdown^8^.

Along with the impact that the pandemic has had over individuals’ everyday lives, contracting COVID-19 itself has also been related to negative mental health outcomes including PTSD^9^, anxiety and depression^10–12^ and psychological distress^13^, along with a range of physical impacts including fatigue, cardiac abnormalities and cognitive impairment^14^. The majority of these mental health outcomes’ studies of COVID-19 survivors have focused on hospitalised individuals at up to 12 month follow up. For example, a large cohort study of just under 154,000 people in the US who experienced COVID-19, showed a modest yet significant association between experiencing COVID-19 infection and psychiatric disorders, persisting at least twelve months^15^. This was found to be worst in individuals admitted to hospital due to COVID-19 but was also evident in non-hospitalised individuals.

There are relatively few studies that have investigated the long-term effects of contracting COVID-19 on later mental health in individuals in the general population who have not been hospitalised due to COVID. One example is a study using a large US sample that found that testing positive for COVID-19 was associated with an increase in psychological distress (measured with the Patient Health Questionnaire (PHQ)) but levels of distress returned to normal soon after the symptoms lessened^16^. Another study using data from the UK Household Longitudinal Study, found elevated levels of psychological distress (assessed using the General Health Questionnaire, GHQ) up to 7 months after testing for probable COVID-19^17^. Most recently, in a study of older adults in the UK, Iob, Steptoe and Zaninotto^18^ found that participants with probable COVID-19 reported significantly poorer mental health up to 6 months later compared to those without probable COVID-19 infection. Therefore, building on this work and by focusing on a nationally representative sample of individuals from the UK general population using a range of indicators of mental health, the current study aimed to increase our understanding of, and potentially inform work exploring the longer-term effects of COVID-19 on mental health.

In this paper, we report data from the UK COVID-19 Mental Health and Wellbeing study, a longitudinal, national survey which ran from March 2020 to July 2021. The present study focuses on contracting suspected or confirmed COVID-19 at the early stages of the pandemic and whether COVID status was associated with mental health outcomes at 1 month (May–June 2020), 5-month (Oct/Nov 2020) and 13-month (June/July 2021) follow up.

## Methods

Participant recruitment was conducted by Taylor McKenzie, a social research company. A non-probability sample of adults (aged 18 years or older) was recruited from across the UK to the UK COVID-19 Mental Health & Wellbeing study (UK COVID-19-MH), with a longitudinal study design. The social research company generated unique identifiers for each of the participants in order to allow responses to be matched throughout the study and to ensure anonymity. UK COVID-19-MH has been detailed previously^5,14^ and was preregistered at AsPredicted.org (#41910).

Between 31st March 2020 and 9th July 2021, members of an existing online UK panel (Panelbase.net) were invited by email to take part in an online survey on health and wellbeing. At wave 1, 7471 panel members were invited to take part and 3077 were included in the final sample (target sample was n = 3,000). A quota sampling methodology was employed, with quotas based on age (18–24 years: 12%; 25–34: 17%; 35–44: 18%; 45–54: 18%; 55–64: 15%; ≥ 65: 20%), gender (women: 51%; men: 49%), socioeconomic grouping (SEG; AB:27%; C1: 28%; C2: 20%; DE: 25%, based on occupation, where A, B and C1 are higher and categories C2, D, E are lower) and region of the UK (12 regions). The quota sampling characteristics were not interlocked. The panel has approximately 300,000 registered adult members and of those invited, 4394 did not take part in the survey. The majority were screened out as a particular quota was full (n = 3527) and the remainder dropped out (n = 867). As outlined above, 3077 participants started and the response rates (%) for each of the subsequent waves were as follows: wave 2 (N = 2742, 89%), wave 3 (N = 2604, 85%), wave 4 (N = 2384, 77%), wave 5 (N = 2144, 70%), wave 6 (N = 2283, 74%), wave 7 (N = 2224, 72%) and wave 8 (1994, 65%).

The first three waves occurred within the first 6 weeks of the UK lockdown (wave 1 (31st March–9th April 2020) wave 2 (10th April–27th April 2020) and wave 3 (28th April–11th May 2020)). The subsequent five waves were conducted over the following 14 months with the interval between waves increasing over time to 7 months (wave 4 (27th May–15th June 2020), wave 5 (17th July–17th August 2020), wave 6 (1st October–4th November 2020) and wave 7 (4th February–2nd March 2021) and wave 8 (2nd June–9th July 2021)). The current analyses focussed on outcomes at waves 4, 6 and 8 for two main reasons. First, we wanted to reduce the number of analytical comparisons for statistical reasons and we were interested in testing the association between COVID-19 status and mental health outcomes over reasonably long time intervals (e.g., wave 6 was 4 months after wave 4 and wave 8 was 7 months after wave 6). Wave 4 (27th May–15th June 2020) coincided with easing of the restrictions while wave 6 (1st October–4th November 2020) coincided with an increase in restrictions again across the UK when cases of COVID-19 were on the rise. Wave 8 (2nd June–9th July 2021) was conducted in summer 2021 which coincided with an easing of government restrictions in all sectors (while COVID secure guidance remained in place). The survey included questions on a wide range of psychological and social measures along with questions about COVID-19.

### Ethical approval

Participants provided written informed consent online. The authors assert that all procedures contributing to this work comply with the ethical standards of the relevant national and institutional committees on human experimentation and with the Helsinki Declaration of 1975, as revised in 2008. The study was approved by the University of Glasgow’s Medical, Veterinary & Life Sciences Ethics Committee (approval number: 200190146) and participants consented for their data to be used in the research. Participants received £1.50 for the completion of each survey and were entered into prize draws.

### Measures

Experience of contracting/having COVID-19 was assessed using a single item measure (“Have you had COVID-19 (coronavirus)?”; “Yes diagnosed and recovered”; “Yes diagnosed and still ill” or “Not formally diagnosed but suspected”; “Don’t know”; “No”).

Depressive symptoms were assessed via the nine-item Patient Health Questionnaire (PHQ-9^19–21^). The 7-item Generalized Anxiety Disorder (GAD-7^22^;) tool was used to assess symptoms of generalised anxiety disorder. Both measures ask how often symptoms have been bothering the respondents in the past 2 weeks on a scale of 0 (not at all) to 3 (nearly every day). Cronbach’s alpha for the scales in the current sample ranged from 0.90 to 0.93, and 0.92 to 0.94, respectively. Scores range from 0 to 27 on the PHQ-9 and 0–21 on the GAD-7, with higher scores indicating higher levels of symptoms of depression and anxiety. Scores of ≥ 10 on both measures are thought to indicate clinically significant cut-offs as indicators of at least moderate levels of depression and anxiety^19,22^.

Mental wellbeing was assessed via the 7-item Short Warwick Edinburgh Mental Wellbeing Scale (SWEMWBS^23^;). Participants were asked to respond about their experiences over the last 2 weeks on a 1 (none of the time) to 5 (all the time) scale. Cronbach’s alphas for the scale in the current sample ranged from 0.89 to 0.92. Scores range from 7 to 35 with a higher score indicating better mental wellbeing. A score of 19.3 is thought to indicate low levels of mental wellbeing and is used as a clinically significant cut-off^23^.

Loneliness was measured using the UCLA 3-item loneliness scale^24^. Participants were asked to respond about their experiences over the last 2 weeks on a three-point scale (hardly ever, some of the time, often). Scores ranged from 3 to 9 where a higher score indicates higher levels of loneliness. Cronbach’s alphas for the scale in the current sample ranged from 0.88 to 0.90. We separated scores based on a cut-off of 7 or more as indicative of high loneliness.

To assess physical and mental health status, participants were asked “Do you have any long-standing physical or mental impairment, illness or disability?” and were then provided a list of physical health conditions (e.g., high blood pressure, diabetes, heart disease, cancer, lung disease etc.) and mental health conditions (e.g., clinically-diagnosed depression, clinically-diagnosed anxiety, attention deficit hyperactivity disorder, neuro-divergent disorders, alcohol/drug problems, another clinically-diagnosed mental health problem). Participants who reported an existing physical or mental health condition received a score of 1 (yes) or 0 (no).

### Statistical analyses

Analyses were conducted in SPSS v.25. Experience of COVID-19 was defined as participants reporting “Yes diagnosed and recovered”; “Yes diagnosed and still ill” or “Not formally diagnosed but suspected” at Wave 1, 2 or 3. Outcomes were scores on the PHQ-9 (depression), GAD-7 (anxiety), SWEMWBS (mental wellbeing) and UCLA-3 (loneliness) at Wave 4 (27th May–15th June 2020); Wave 6 (1st October–4th November 2020) and Wave 8 (2nd June–9th July 2021). Initially, Chi square analyses were conducted to assess any differences in demographics in individuals reporting probable COVID-19. Next, hierarchical linear regression analyses were conducted to assess the relationship between COVID-19 status and mental health outcomes. In the these analyses, in order to control for their effects, gender (female, male), age (under and equal to 30, over 30), physical health status (any physical health conditions reported: no/yes), ethnicity (White vs minority ethnic groups) (socioeconomic group (SEG): high (A + B + C1) vs. low (C2 + D + E)) were entered in step 1, followed by probable COVID infection (yes/no) at step 2, and mental health status (presence of mental health conditions: yes/no) at step 3. An interaction term between experience of COVID-19 and mental health status was then calculated (and entered in step 4) in order to assess the potential moderating effect of mental health status on the COVID-mental health outcome relationship. These analyses were repeated using hierarchical logistic regressions using the clinically meaningful cut-offs for each of the outcomes. Again, in order to control for their effects, gender, age, ethnicity, physical health and SEG were entered in step 1, followed by probable COVID infection at step 2, mental health status at step 3 and the probable COVID-19 status by mental health status interaction. Finally, we also explored whether there was a bidirectional relationship between having an existing mental health condition and contracting probable COVID-19 infection (yes/no) at any stage during waves 1 to 8. Using hierarchical logistic regression, the control variables (gender, age, ethnicity physical health & SEG) were entered at step 1 and mental health status at step 2.

Multiple imputation was also carried out and 10 imputed datasets were created; analyses were then conducted on a randomly selected dataset and the results of the imputed and unimputed datasets were compared following the same method used in O’Connor et al.^7^. There were no differences in results found between the imputed and non-imputed datasets.

## Results

Just under 9% of participants reported COVID-19 at wave 1 (8.7%; n = 268), 8.5% reported COVID-19 at wave 2 (n = 234) and 9.1% reported COVID-19 at wave 3 (N = 237). In total, 393 (12.8%) participants reported COVID-19 at waves 1, 2 or 3. Across the overall sample, depression and anxiety scores were highest at Wave 6 and lowest at Wave 8, mental wellbeing scores were lowest at Wave 6 and highest at Wave 8.

**Figure 1:**
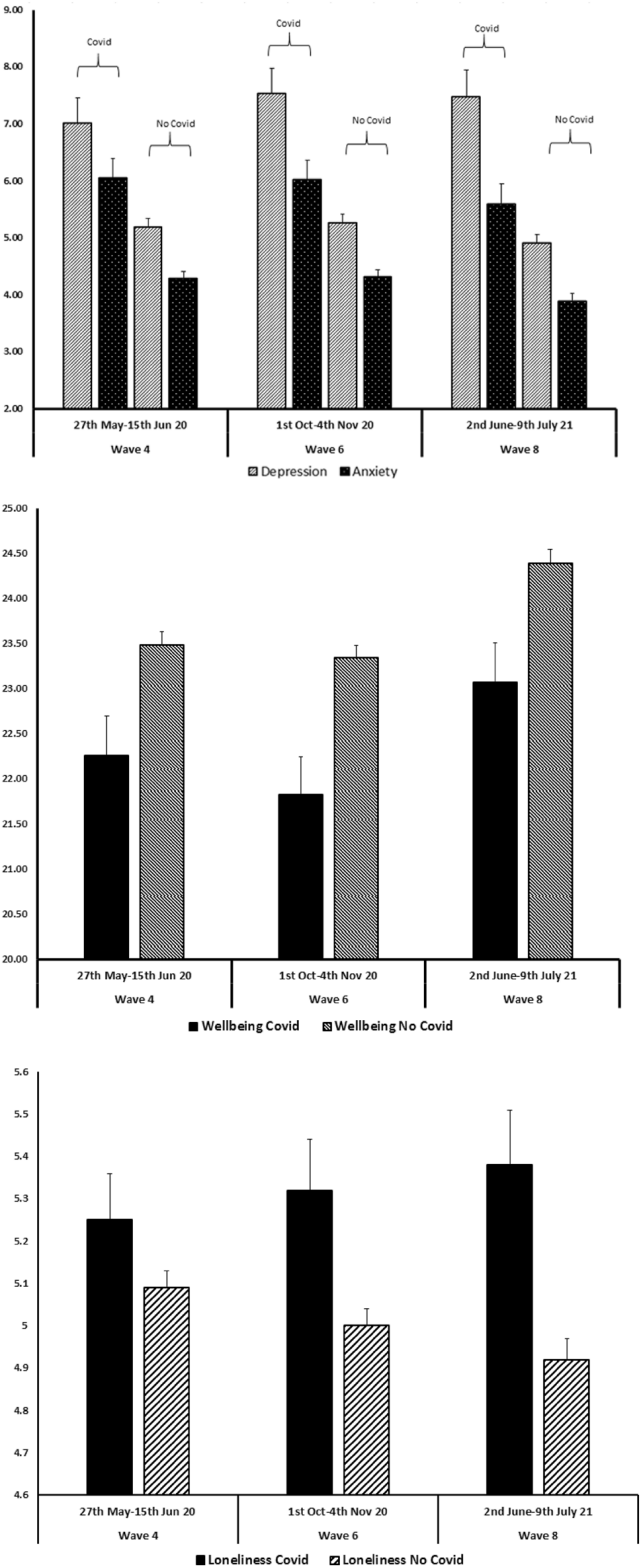
Mean mental health scores (top panel: depression and anxiety; middle panel: wellbeing; bottom panel: loneliness) at 1 month (May/June 2020), 5 month (Oct/Nov 2020) and 13 month (June/July 2021) follow up in individuals with and without COVID-19 at waves 1, 2 or 3.

Individuals who reported that they had contracted probable COVID-19 had poorer outcomes on all three mental health measures with higher depression, anxiety and loneliness scores, and lower wellbeing (see Fig. 1 & Supplementary Table 1) compared to those who did not report contracting probable COVID-19. Chi square analyses were conducted to assess any differences in demographics in individuals reporting COVID-19. Males were more likely to report they had COVID-19 (14.5%; N = 200) compared to females (11.4%; N = 193), Χ^2^ (df = 1) = 6.45, *p* = 0.01, however there were no age or SEG differences found.

### Associations between probable COVID-19 infection and subsequent mental health outcomes

Regression analyses (Tables 1; 2) were conducted, with age, gender, ethnicity, SEG and physical health status entered at step 1 and the binary COVID-19 variable (whether or not COVID-19 was reported at Waves 1, 2 or 3) entered in step 2, mental health status (presence of any pre-existing conditions) was entered at step 3 and the interaction between COVID-19 and mental health status was entered at step 4. The results of these analyses demonstrated that across all three time points and when controlling for a range of demographic variables and the presence of pre-existing mental health conditions (see step 3), probable COVID-19 was found to be related to poorer mental health (depression wave 4: β = 1.37, SE = 0.34, *p* < 0.001; wave 6: β = 1.60, SE = 0.39, *p* < 0.001; wave 8: β = 2.36, SE = 0.42, *p* < 0.001; anxiety wave 4: β = 1.38, SE = 0.29, *p* < 0.001; wave 6: β = 1.29, SE = 0.32, *p* < 0.001; wave 8: β = 1.69, SE = 0.37, *p* < 0.001; wellbeing wave 4: β = −1.17, SE = 0.39, *p* = 0.003; wave 6: β = −0.78, SE = 0.47, *p* = 0.059; wave 8: β = −1.03, SE = 0.53, *p* = 0.028). Probable COVID-19 was found to be related to loneliness at wave 8 only (β = 0.36, SE = 0.14, *p* = 0.008). Finally, there were no moderating effects of mental health status on the relationship between COVID-19 and mental health outcomes (see step 4). It is important to note that having a pre-existing mental health condition was significantly associated with all of the mental health outcomes at each wave indicating that the associations between probable COVID-19 infection and mental health condition status were independent.

**Table 1:** Hierarchical regression analyses investigating the associations between probable COVID-19 infection during waves 1–3 (Mar-May 2020) and depression and anxiety at 1 month (May/June 2020), 5 month (Oct/Nov 2020) and 13 month (June/July 2021) follow up waves.

		Wave 4 May–June 2020			Wave 6 Oct–Nov 2020			Wave 8 June-July 2021		
		B	SE	*p* value	B	SE	*p* value	B	SE	*p* value
**Depression**										
Step 1	Gender	− 1.59	0.25	< 0.001	− 1.84	0.28	< 0.001	− 1.43	0.30	< 0.001
	SEG	0.81	0.25	0.001	0.98	0.28	< 0.001	1.02	0.30	< 0.001
	Age	− 2.14	0.31	< 0.001	− 2.56	0.36	< 0.001	− 1.60	0.40	< 0.001
	Ethnicity	0.16	0.46	0.733	− 0.44	0.54	0.419	0.94	0.58	0.107
	Physical health	1.12	0.27	< 0.001	1.31	0.30	< 0.001	1.28	0.32	< 0.001
Step 2	Gender	− 1.64	0.25	< 0.001	− 1.87	0.28	< 0.001	− 1.48	0.29	< 0.001
	SEG	0.85	0.25	< 0.001	1.04	0.28	< 0.001	1.05	0.29	< 0.001
	Age	− 2.05	0.31	< 0.001	− 2.50	0.36	< 0.001	− 1.53	0.40	< 0.001
	Ethnicity	− 0.07	0.46	0.873	− 0.36	0.54	0.506	1.01	0.58	0.080
	Physical health	1.11	0.27	< 0.001	1.31	0.30	< 0.001	1.29	0.31	< 0.001
	COVID	1.77	0.37	< 0.001	2.15	0.42	< 0.001	2.76	0.45	< 0.001
Step 3	Gender	− 1.06	0.23	< 0.001	− 1.27	0.26	< 0.001	− 1.00	0.28	< 0.001
	SEG	0.32	0.23	0.14	0.49	0.26	0.060	0.67	0.28	0.015
	Age	− 1.53	0.29	< 0.001	− 2.09	0.33	< 0.001	− 1.01	0.38	0.007
	Ethnicity	0.33	0.42	0.434	0.12	0.50	0.806	1.40	0.54	0.010
	Physical health	0.67	0.25	0.008	0.85	0.28	0.002	0.86	0.30	0.004
	COVID	1.37	0.34	< 0.001	1.60	0.39	< 0.001	2.36	0.42	< 0.001
	Mental health status	5.21	0.26	< 0.001	5.39	0.30	< 0.001	5.08	0.32	< 0.001
Step 4	Gender	− 1.06	0.23	< 0.001	− 1.28	0.26	< 0.001	− 1.00	0.28	< 0.001
	SEG	0.32	0.23	0.164	0.49	0.26	0.057	0.67	0.28	0.015
	Age	− 1.52	0.29	< 0.001	− 2.07	0.33	< 0.001	− 0.99	0.38	0.009
	Ethnicity	0.34	0.42	0.422	0.14	0.50	0.778	1.42	0.54	0.009
	Physical health	0.67	0.25	0.008	0.85	0.28	0.002	0.86	0.30	0.004
	COVID	1.53	0.41	< 0.001	2.00	0.48	< 0.001	2.63	0.50	< 0.001
	Mental health status	5.77	0.88	< 0.001	6.78	1.00	< 0.001	6.11	1.09	< 0.001
	COVID* MH	− 0.49	0.73	0.51	− 1.23	0.84	0.143	− 0.90	0.91	0.323
**Anxiety**										
Step 1	Gender	− 1.61	0.22	< 0.001	− 1.87	0.23	< 0.001	− 1.44	0.24	< 0.001
	SEG	0.56	0.21	0.009	0.50	0.23	0.029	0.44	0.24	0.068
	Age	− 1.84	0.27	< 0.001	− 1.83	0.30	< 0.001	− 1.86	0.33	< 0.001
	Ethnicity	− 0.49	0.39	0.213	− 0.23	0.44	0.605	− 0.01	0.48	0.977
	Physical health	0.67	0.23	0.004	0.81	0.25	< 0.001	0.87	0.26	< 0.001
Step 2	Gender	− 1.65	0.21	< 0.001	− 1.90	0.23	< 0.001	− 1.47	0.24	< 0.001
	SEG	0.60	0.21	< 0.005	0.55	0.23	0.016	0.46	0.24	< 0.056
	Age	− 1.76	0.26	< 0.001	− 1.78	0.30	< 0.001	− 1.81	0.33	< 0.001
	Ethnicity	− 0.41	0.39	0.296	− 0.16	0.44	0.709	0.03	0.47	0.950
	Physical health	0.66	0.23	0.004	0.81	0.24	< 0.001	0.88	0.26	< 0.001
	COVID	1.72	0.31	< 0.001	1.76	0.35	< 0.001	1.69	0.37	< 0.001
Step 3	Gender	− 1.17	0.20	< 0.001	− 1.38	0.21	< 0.001	− 1.07	0.23	< 0.001
	SEG	0.15	0.20	0.440	0.07	0.21	0.751	0.14	0.23	0.527
	Age	− 1.32	0.25	< 0.001	− 1.43	0.27	< 0.001	− 1.38	0.31	< 0.001
	Ethnicity	− 0.07	0.36	0.849	0.25	0.41	0.537	0.36	0.44	0.416
	Physical health	0.29	0.22	0.183	0.41	0.23	0.070	0.51	0.24	0.033
	COVID	1.38	0.29	< 0.001	1.29	0.32	< 0.001	1.36	0.34	< 0.001
	Mental health status	4.37	0.23	< 0.001	4.67	0.24	< 0.001	4.27	0.26	< 0.001
Step 4	Gender	− 1.17	0.20	< 0.001	− 1.38	0.21	< 0.001	− 1.07	0.23	< 0.001
	SEG	0.15	0.20	0.438	0.07	0.21	0.743	0.14	0.23	0.526
	Age	− 1.31	0.25	< 0.001	− 1.41	0.25	< 0.001	− 1.37	0.31	< 0.001
	Ethnicity	− 0.06	0.36	0.870	0.26	0.41	0.526	0.36	0.44	0.409
	Physical health	0.29	0.22	0.183	0.41	0.23	0.070	0.51	0.24	0.030
	COVID	1.56	0.35	< 0.001	1.45	0.39	< 0.001	1.44	0.41	< 0.001
	Mental health status	4.99	0.75	< 0.001	5.23	0.82	< 0.001	4.59	0.89	< 0.001
	COVID* MH	− 0.54	0.63	0.388	− 0.49	0.68	0.471	− 0.28	0.75	0.707

MH = Mental health status; COVID status = yes (1) or no (0); gender = female (1), male (2); age group = less than or equal to 30 (1) or over 30 (2); ethnicity = White (1) or minority ethnic groups (2); socioeconomic group = high (1) or low (2); physical health conditions reported = no (0) or yes (1), mental health conditions reported: no (0) or yes (1). See Supplementary Notes for Table 1.

**Table 2:** Hierarchical regression analyses investigating the associations between probable COVID-19 infection during waves 1–3 (Mar–May 2020) and wellbeing and loneliness at 1 month (May/June 2020), 5 month (Oct/Nov 2020) and 13 month (June/July 2021) follow up waves.

		Wave 4 May–June 2020			Wave 6 Oct–Nov 2020			Wave 8 June–July 2021		
		B	SE	*p* value	B	SE	*p* value	B	SE	*p* value
**Wellbeing**										
Step 1	Gender	0.94	0.27	< 0.001	1.19	0.29	< 0.001	0.88	0.32	0.006
	SEG	− 0.82	0.27	0.002	− 1.03	0.28	< 0.001	− 0.90	0.32	0.005
	Age	2.07	0.33	< 0.001	2.11	0.37	< 0.001	2.00	0.44	< 0.001
	Ethnicity	− 0.43	0.49	0.383	− 0.61	0.55	0.271	− 0.96	0.63	0.128
	Physical health	− 0.21	0.29	0.475	− 0.20	0.30	0.522	− 0.13	0.34	0.698
Step 2	Gender	0.97	0.27	< 0.001	1.20	0.29	< 0.001	0.90	0.32	0.005
	SEG	− 0.85	0.27	0.001	− 1.06	0.28	< 0.001	− 0.92	0.32	0.004
	Age	2.01	0.33	< 0.001	2.07	0.37	< 0.001	1.96	0.44	< 0.001
	Ethnicity	− 0.48	0.49	0.325	− 0.65	0.55	0.236	− 1.00	0.63	0.114
	Physical health	− 0.20	0.29	0.493	− 0.19	0.30	0.527	− 0.14	0.34	0.685
	COVID	− 1.17	0.39	0.003	− 1.25	0.43	0.004	− 1.36	0.49	0.005
Step 3	Gender	0.46	0.26	0.076	0.69	0.27	0.011	0.51	0.31	0.105
	SEG	− 0.37	0.25	0.141	− 0.58	0.27	0.032	− 0.61	0.31	0.049
	Age	1.54	0.32	< 0.001	1.72	0.35	< 0.001	1.54	0.42	< 0.001
	Ethnicity	− 0.84	0.47	0.434	− 1.06	0.52	0.042	− 1.32	0.61	0.031
	Physical health	0.19	0.28	0.484	0.20	0.29	0.486	0.21	0.33	0.518
	COVID	− 0.81	0.38	0.031	− 0.78	0.41	0.059	− 1.03	0.47	0.028
	Mental health status	− 4.62	0.29	< 0.001	− 4.61	0.31	< 0.001	− 4.15	0.36	< 0.001
Step 4	Gender	0.46	0.26	0.076	0.70	0.27	0.011	0.51	0.31	0.103
	SEG	− 0.38	0.25	0.140	− 0.59	0.27	0.030	− 0.61	0.31	0.049
	Age	1.52	0.32	< 0.001	1.71	0.35	< 0.001	1.53	0.42	< 0.001
	Ethnicity	− 0.86	0.47	0.065	− 1.07	0.52	0.040	− 1.33	0.61	0.029
	Physical health	0.20	0.28	0.482	0.20	0.29	0.486	0.21	0.33	0.520
	COVID	− 1.18	0.45	0.009	− 1.07	0.50	0.033	− 1.20	0.56	0.033
	Mental health status	− 5.96	0.97	< 0.001	− 5.64	1.05	< 0.001	− 4.80	1.22	< 0.001
	COVID*MH	1.17	0.81	0.148	0.90	0.88	0.306	0.57	1.03	0.576
**Loneliness**										
Step 1	Gender	− 0.53	0.08	< 0.001	− 0.55	0.09	< 0.001	− 0.48	0.09	< 0.001
	SEG	0.26	0.08	< 0.001	0.28	0.09	0.001	0.27	0.09	0.004
	Age	− 0.47	0.10	0.001	− 0.52	0.11	< 0.001	− 0.44	0.13	< 0.001
	Ethnicity	0.03	0.15	0.867	0.08	0.17	0.623	0.13	0.18	0.464
	Physical health	0.04	0.09	0.672	0.07	0.09	0.421	0.12	0.10	0.240
Step 2	Gender	− 0.54	0.32	< 0.001	− 0.56	0.09	< 0.001	− 0.49	0.09	< 0.001
	SEG	0.26	0.08	< 0.001	0.29	0.09	< 0.001	0.28	0.09	0.003
	Age	− 0.46	0.10	< 0.001	− 0.51	0.11	< 0.001	− 0.42	0.13	< 0.001
	Ethnicity	0.03	0.15	0.826	0.09	0.17	0.578	0.15	0.18	0.425
	Physical health	0.04	0.09	0.682	0.07	0.09	0.425	0.12	0.10	0.231
	COVID	0.16	0.12	0.166	0.29	0.13	0.025	0.45	0.14	0.001
Step 3	Gender	− 0.42	0.08	< 0.001	− 0.44	0.08	< 0.001	− 0.39	0.09	< 0.001
	SEG	0.15	0.08	< 0.049	0.18	0.08	0.034	0.20	0.09	0.031
	Age	− 0.35	0.10	< 0.001	-0.43	0.11	< 0.001	− 0.32	0.12	0.011
	Ethnicity	0.12	0.14	0.415	0.19	0.16	0.240	0.23	0.18	0.197
	Physical health	− 0.06	0.08	0.504	-0.02	0.09	0.824	0.03	0.10	0.782
	COVID	0.08	0.11	0.484	0.18	0.13	0.153	0.36	0.14	0.008
	Mental health status	1.08	0.09	< 0.001	1.09	0.10	< 0.001	1.08	0.11	< 0.001
Step 4	Gender	− 0.42	0.08	< 0.001	− 0.44	0.08	< 0.001	− 0.39	0.09	< 0.001
	SEG	0.15	0.08	0.049	0.18	0.08	0.035	0.20	0.09	0.031
	Age	− 0.35	0.10	< 0.001	− 0.43	0.11	< 0.001	− 0.32	0.12	0.011
	Ethnicity	0.12	0.14	0.403	0.19	0.16	0.243	0.23	0.18	0.200
	Physical health	− 0.06	0.08	0.504	− 0.02	0.09	0.824	0.03	0.10	0.783
	COVID	0.13	0.14	< 0.001	0.16	0.16	0.293	0.35	0.16	0.035
	Mental health status	1.27	0.30	< 0.001	1.03	0.32	0.002	1.02	0.36	0.004
	COVID* MH	− 0.16	0.25	0.516	0.06	0.27	0.829	0.06	0.30	0.846

MH = Mental health status; COVID status = yes (1) or no (0); gender = female (1), male (2); age group = less than or equal to 30 (1) or over 30 (2); ethnicity = White (1) or minority ethnic groups (2); socioeconomic group = high (1) or low (2); physical health conditions reported = no (0) or yes (1), mental health conditions reported: no (0) or yes (1). See Supplementary Notes for Table 2.

### Associations between probable COVID-19 infection and subsequent clinically meaningful cut-offs for mental health outcomes

**Figure 2:**
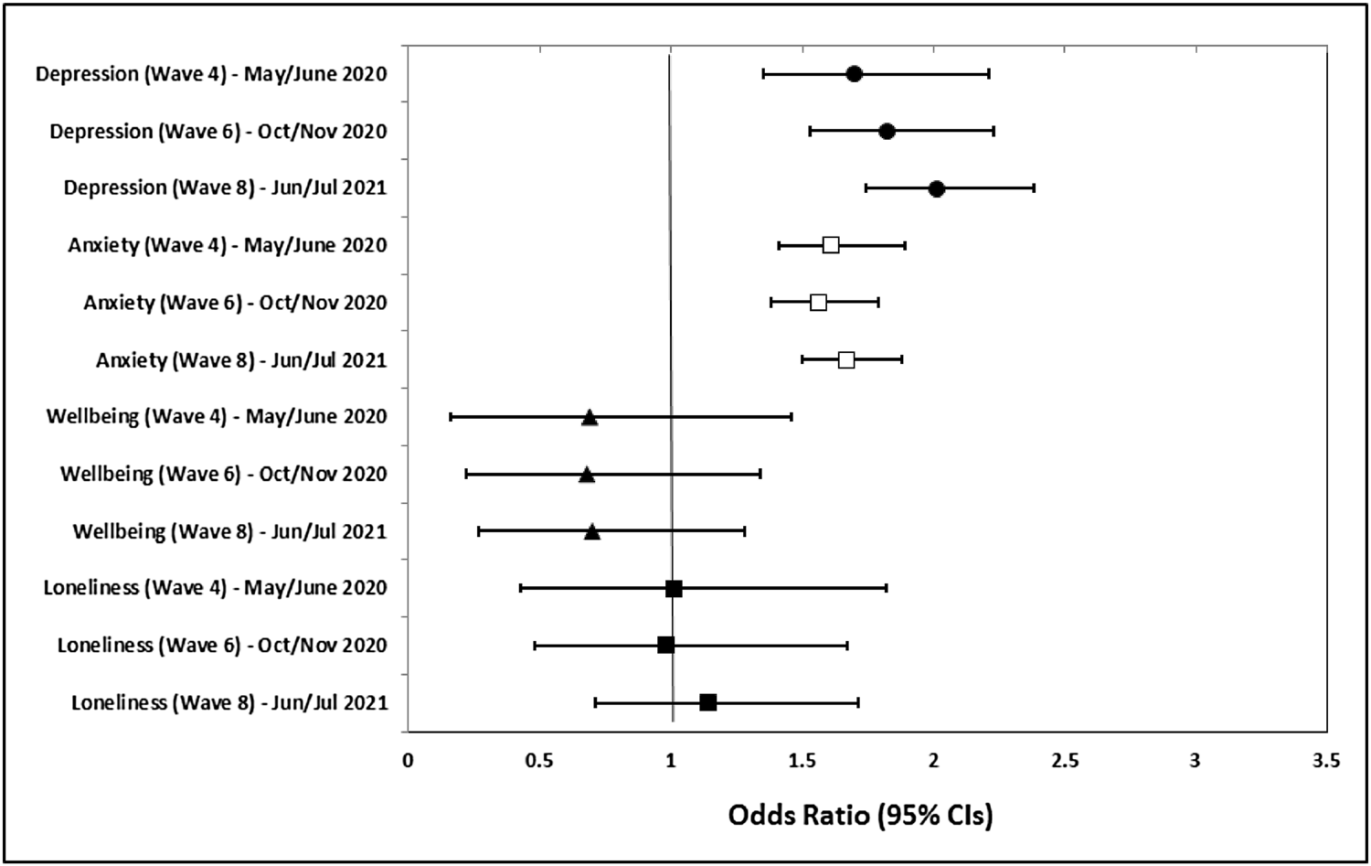
Associations between reporting probable COVID-19 infection during first 6 weeks of the UK national lockdown (Mar/May 2020) and clinically meaningful cut-offs for mental health outcomes at 1 month (May/June 2020), 5 month (Oct/Nov 2020) and 13 month (June/July 2021) follow up waves.

As outlined above, the analyses were repeated using the clinically meaningful cut-offs (see Supplementary Table 2). The results of these analyses showed that across all three time points and when controlling for the demographic variables and pre-existing mental health conditions, probable COVID-19 infection was associated with a greater odds of reporting clinically meaningful levels of depression (wave 4: OR = 1.70, 95% CI 1.27, 2.27, *p* < 0.001; wave 6: OR = 1.82, 95% CI 1.32, 2.51, *p* < 0.001; wave 8: OR = 2.01, 95% CI 1.43, 2.82, *p* < 0.001), anxiety (wave 4: OR = 1.61, 95% CI 1.18, 2.19, p = 0.002; wave 6: OR = 1.56, 95% CI 1.10, 2.22, *p* = 0.013; wave 8: OR = 1.67, 95% CI 1.14, 2.44, *p* = 0.008) and wellbeing (wave 4: OR = 0.69, 95% CI 0.52, 0.90, *p* = 0.007; wave 6: OR = 0.68, 95% CI 0.50, 0.91, *p* = 0.010; wave 8: OR = 0.70, 95% CI 0.50, 0.98, *p* = 0.04). Probable COVID-19 was not significantly related to clinically meaningful levels of loneliness (see Fig. 2 and Supplementary Tables 3 and 4).

With the exception of anxiety levels at wave 4 (May/June 2020), there were no moderating effects of mental health status on the relationship between COVID-19 and mental health cut-off. When the wave 4 interaction for anxiety was decomposed, the analyses found that probable COVID-19 infection, compared to not having probable COVID-19 infection, was associated with greater odds of reporting clinically meaningful levels of anxiety in May/June 2020 in individuals without an existing mental health condition (OR = 2.40, 95% CI 1.62, 3.56, *p* < 0.001) but not in individuals with an existing mental health condition (OR = 0.99, 95% CI 0.62, 1.57, *p* = 0.96).

Finally, we conducted a logistic regression to test whether there was a bidirectional relationship between having an existing mental health condition at the beginning of the study and contracting probable COVID-19 infection at any stage during the study. The results of this analysis (controlling for age, gender, socioeconomic status, ethnicity and pre-existing physical health condition) found that having a pre-existing mental health condition was associated with greater odds of having probable COVID-19 over the following 13 months (OR = 1.31, 95% CI 1.05, 1.63, *p* = 0.016).

## Discussion

The current study aimed to investigate whether contracting suspected or confirmed COVID-19 at the early stages of the pandemic predicted mental health outcomes at 1-month, 5-month and 13-month follow up. Around 9% of participants reported contracting COVID-19 between March and May 2020, with just under 13% of the overall sample reporting COVID-19 at any one of the three time points. Contracting COVID-19 was associated with poorer mental health outcomes with these effects lasting up to 13 months (July 2021). Reporting COVID-19 early in the pandemic was also associated with loneliness at the latest time point only. Moreover, when investigating clinical cut-offs for the mental health outcomes, probable COVID-19 infection was found to be associated with a greater odds of reporting clinically meaningful levels of depression, anxiety and poor wellbeing.

Previous studies have found associations between contracting COVID and increased reporting of psychiatric illnesses in the US^15^. This along with the results of the present study support the idea that contracting COVID-19 is associated with both physical^14,25^ and mental health impairments. Previous work has demonstrated that COVID-19 is related to increased incidence of anxiety and depression^10–12^ and psychological distress^13^. The present study demonstrates that these findings are also generalisable to non-hospitalised individuals and are still observable at up to 13-months follow up. In addition, the current findings are broadly consistent with and extend two other studies conducted in the UK. Niedzwiedz et al.^17^, using data from the UK Household Longitudinal Study, found elevated levels of psychological distress up to 7 months after testing for probable COVID-19. Similarly, Iob, Steptoe and Zaninotto^18^, in a study of UK older adults, found that participants with probable COVID-19 reported significantly poorer mental health up to 6 months later compared to those without probable COVID-19 infection.

It is also interesting to note that, although the amount of explained variance (in the hierarchical linear regressions) is relatively small in the current study, the odds ratios for the associations between probable COVID infection and anxiety and depression at 5 months follow-up (Oct/Nov 2020, OR_depression_ = 1.82; OR_anxiety_ 1.56) are notable and very similar to odds ratios for anxiety and depression at 7 months (Nov/Dec 2020: OR_depression_ = 1.56; OR_anxiety_ = 1.55) reported by Iob, Steptoe and Zaninotto^18^. Taken together, these findings suggest that the odds of experiencing clinically meaningful levels of depression and anxiety in the medium term (~ 6 months later) are around 1.6 times (~ OR = 1.6) higher following probable COVID-19 infection than not having probable COVID-19 infection. Moreover, the current study also demonstrates that the odds of experiencing clinically meaningful levels of depression and anxiety in the longer term (~ 13 months later) are 2.01 times and 1.67 times higher (respectively) following COVID-19 infection (July 2021, OR_depression_ = 2.01; OR_anxiety_ = 1.67).

The current study also found that having a pre-existing mental health condition at the beginning of the pandemic was related to increased odds of contracting probable COVID infection over the following 13 months. This finding is consistent with Taquet et al.^10^, who found, in a large retrospective cohort study in the US, that having a psychiatric diagnosis in the previous year was associated with a higher incidence of COVID-19 diagnosis. Another earlier study, using a case–control design, also found that having a psychiatric diagnosis was associated with higher odds of being diagnosed with COVID-19^26^. Several plausible explanations have been suggested for this association such as behavioural (e.g., being less adherent to COVID-19 guidance and restrictions and lifestyle factors (e.g., smoking) or biological vulnerabilities associated with the psychiatric condition or psychotropic medications^10^. However, importantly, in our analyses, the associations between probable COVID-19 and subsequent mental health outcomes remained statistically significant even when pre-existing mental health condition status was included in the analyses demonstrating that mental health status was not accounting for the observed associations.

What mechanisms may account for the observed associations between contracting probable COVID-19 infection and subsequent mental health outcomes? It is likely that multiple factors play a role and different factors will have been more influential at different stages of the pandemic. For example, Iob, Steptoe and Zaninotto^18^ showed that financial stressors were associated with probable COVID-19 infection in June/July 2020 but not in November/December 2020. Evidence from studies of patients with long Covid and studies of participants reporting symptoms lasting ≥ 28 days has identified the importance of post-viral symptoms such as fatigue, sleep disturbance, headaches, cognitive impairment, loss of taste and smell, breathlessness and post-exertional malaise^13,27^. Therefore, it is likely that the experience of similar symptoms, and their associated underlying physiological perturbations, in individuals in the general population following contracting COVID-19 may contribute to high levels of depression, anxiety and poor wellbeing. Psychological factors and the existence of government restrictions are also likely to play a role. For example, higher levels of COVID-19 related worry and information seeking have been shown to be associated with clinically meaningful levels of anxiety and depression in this cohort already^7,28^. Further investigation is required in order to understand the precise causal mechanisms that account for the association between COVID-19 infection and poor mental health outcomes.

With the exception of one mental health outcome at a single time point (anxiety levels in May/June 2020), the present study also demonstrated that the mental health status of participants did not moderate the relationship between contracting COVID-19 and subsequent mental health, suggesting that the relationship remains regardless of whether individuals had pre-existing mental health conditions. This is in contrast to our previous work^7^ where mental health status significantly moderated the relationship between both COVID-related worry and rumination and mental health outcomes along with other work which has suggested that the pandemic has had a greater negative impact on individuals with pre-existing mental health conditions^29,30^. Nevertheless, these findings are consistent with a recent meta-analysis comparing mental health before and during the pandemic that did not find evidence of more pronounced effects on mental health symptoms in individuals with a pre-existing mental health condition^2^. However, the latter meta-analysis did not explore the interaction between contracting COVID-19, mental health status, and longer-term mental health outcomes. It is also interesting to note that the only moderating effect that we did observe was in the opposite direction to what we would have expected, such that contracting COVID-19, compared to not contracting COVID-19, was associated with greater anxiety levels in individuals who did not have an existing mental health condition. It is difficult to reconcile this singular result and given that no other moderating effects were observed for any other mental health outcomes, it is likely this may be a chance finding.

The current findings have implications for public health policy and mental health interventions as we continue to emerge from the COVID-19 pandemic. If we extrapolate beyond the UK COVID-19 Mental Health and Wellbeing Study, it is clear that following COVID infection, there will be a relatively large number of individuals who are likely to continue experiencing clinically meaningful levels of depression and anxiety, possibly alongside other post-viral symptoms such as fatigue and cognitive impairment. Therefore, the current findings highlight the importance for general practitioners and other healthcare professionals to put in place treatments and support for mental health, as well as physical health, for symptomatic patients who have contracted COVID-19 infection.

Strengths of the present study include its relatively large, quota-based, and longitudinal sample which enabled the monitoring of mental health up to 13 months following initial questioning. However, there are also limitations of the study sampling. One particular limitation is that participants that had contracted COVID-19 along with those reporting poor mental health outcomes may have been less likely to respond to the questionnaires. In addition to this, despite there being good evidence supporting the validity of self-reporting COVID-19^31,32^, the present study relies on participants self-reporting their COVID-19 status at a time when there was limited testing taking place and therefore it is difficult to know the accuracy of these self-reports. Nevertheless, it is important to note that the levels of probable COVID-19 infection reported in the current study are similar to two other key UK studies conducted at a similar time (8.9%^17^; 9.7%^18^). We also recognise that our measure of existing mental health status is self-reported and not an objective clinical diagnosis corroborated using patient records. This approach was chosen for pragmatic reasons given the speed that was required to launch this large scale, nationally representative study shortly after the UK entered lockdown.

In conclusion, the present study demonstrated that experiencing COVID-19 during the early stages of the pandemic was associated with poorer mental health outcomes (depression, anxiety and poor wellbeing) and loneliness up to 13-months after initial questioning. The current study demonstrates that contracting probable COVID-19 was related to long-lasting associations with mental health and that the relationship between mental health status and probable COVID-19 is bidirectional.

## Supplementary Information

Supplementary Information.

## Data Availability

The data that support the findings of this study are available from the corresponding author (D.O’C.), upon reasonable request.
