# Supplementary material for: Probable COVID-19 infection is associated with subsequent poorer mental health and greater loneliness in the UK COVID-19 Mental Health and Wellbeing study
Source: Sci Rep. 2022 Dec 2;12:20795. doi: 10.1038/s41598-022-24240-3 (PMC9718764; doi:10.1038/s41598-022-24240-3)
Supplement: Supplementary file 1 — Supplementary Information. [file 41598_2022_24240_MOESM1_ESM.docx]

| Supplementary Table 1. Mean mental health and loneliness scores by COVID-19 versus no COVID-19 status at 1 month (May/June 2020), 5 month (Oct/Nov 2020) and 13 month (June/July 2021) follow up waves | | | | | | | | | | | | |  |
| --- | --- | --- | --- | --- | --- | --- | --- | --- | --- | --- | --- | --- | --- |
|  |  | Wave 4 (N = 2384)  May/June 2020 | | | | Wave 6 (N = 2283)  Oct/Nov 2020 | | | | Wave 8 (N = 1994)  June/July 2021 | | | |
|  |  | Depression | Anxiety | Wellbeing | Loneliness | Depression | Anxiety | Wellbeing | Loneliness | Depression | Anxiety | Wellbeing | Loneliness |
| No COVID-19 | Mean (SD) | 5.20 (5.97) | 4.30 (5.08) | 23.49 (6.40) | 5.09 (1.93) | 5.27 (6.24) | 4.32 (5.18) | 23.34 (6.37) | 5.00 (1.95) | 4.91 (6.12) | 3.90 (5.08) | 24.39 (6.74) | 4.92 (1.95) |
| COVID-19 | Mean (SD) | 7.02 (6.64) | 6.06 (5.91) | 22.26 (6.76) | 5.25 (1.96) | 7.54 (7.37) | 6.03 (5.67) | 21.83 (6.95) | 5.32 (1.93) | 7.48 (7.32) | 5.60 (5.46) | 23.07 (6.78) | 5.38 (2.00) |

| Supplementary Table 2. Number of participants scoring above and below the cut-offs for mental health and loneliness scores by COVID-19 versus no COVID-19 status at 1 month (May/June 2020), 5 month (Oct/Nov 2020) and 13 month (June/July 2021) follow up waves | | | | | | | | | | | | |  |
| --- | --- | --- | --- | --- | --- | --- | --- | --- | --- | --- | --- | --- | --- |
|  |  | Wave 4 (N = 2384)  May/June 2020 | | | | Wave 6 (N = 2283)  Oct/Nov 2020 | | | | Wave 8 (N = 1994)  June/July 2021 | | | |
|  |  | Depression | Anxiety | Wellbeing | Loneliness | Depression | Anxiety | Wellbeing | Loneliness | Depression | Anxiety | Wellbeing | Loneliness |
| **No COVID-19** |  |  |  |  |  |  |  |  |  |  |  |  |  |
| Below cut-off | N (%) | 1657 (79.5) | 1751 (84.0) | 504 (24.2) | 1667 (80.0) | 1595 (79.5) | 1694 (84.4) | 524 (26.1) | 1620 (80.8) | 1410 (80.4) | 1507 (85.9) | 391 (22.3) | 1430 (81.5) |
| Above cut-off | N (%) | 428 (20.5) | 334 (16.0) | 1581 (75.8) | 418 (20.0) | 411 (20.5) | 312 (15.6) | 1482 (73.9) | 386 (19.2) | 344 (19.6) | 247 (14.1) | 1363 (77.7) | 324 (18.5) |
| **COVID-19** |  |  |  |  |  |  |  |  |  |  |  |  |  |
| Below cut-off | N (%) | 204 (68.2) | 224 (74.9) | 100 (33.4) | 234 (78.3) | 184 (66.4) | 211 (76.2) | 103 (37.2) | 215 (77.6) | 165 (68.8) | 185 (77.1) | 71 (29.6) | 1.84 (76.7) |
| Above cut-off | N (%) | 95 (31.8) | 75 (25.1) | 199 (50.6) | 65 (21.7) | 93 (33.6) | 66 (23.8) | 174 (62.8) | 62 (22.4) | 75 (31.3) | 55 (22.9) | 169 (70.4) | 56 (23.3) |

Supplementary notes for **Table 1**

*Depression* Wave 4; Step 1: Adj R^2^ = .05; F(5, 2371) = 25.94, p<.001; Step 2: Adj R^2^ = .06, F(1, 2370) = 23.36, p<.001; Step 3: Adj R^2^ = .19, F(1, 2369) = 393.68, p<.001; Step 4: Adj R^2^ = .19, F(1, 2368) = .44, p=.508; Wave 6 Step 1: Adj R^2^ = .06; F(5, 2057) = 28.05, p<.001; Step 2: Adj R^2^ = .07, F(1, 2056) = 25.92, p<.001; Step 3: Adj R^2^ = .20, F(1, 2055) = 330.38, p<.001; Step 4: Adj R^2^ = .20, F(1, 2055) = 330.38, p<.001; Wave 8; Step 1: Adj R^2^ = .04; F(5, 1773) = 14.48, p<.001; Step 2: Adj R^2^ = .06, F(1, 1772) = 38.32, p<.001; Step 3: Adj R^2^ = .17, F(1, 1771) = 252.69, p<.001; Step 4: Adj R^2^ = .17, F(1, 1770) = 0.98, 0.323. *Anxiety* Wave 4; Step 1: Adj R^2^ = .06 F(5, 2371) = 28.51, p<.001; Step 2: Adj R^2^ = .07, F(1, 2370) = 30.01, p<.001; Step 3: Adj R^2^ = .19, F(1, 2369) = 374.87, p<.001; Step 4: Adj R^2^ = .19, F(1, 2368) = 0.75, p=.38; Wave 6 Step 1: Adj R^2^ = .06; F5, 2057) =27.86, p<.001; Step 2: Adj R^2^ = .07, F(1, 2056) = 25.86, p<.001; Step 3: Adj R^2^ = .21, F(1, 2055) = 372.73, p<.001; Step 4: Adj R^2^ = .21, F(1, 2054) = 0.52, p=.47; Wave 8; Step 1: Adj R^2^ = .05; F(5, 1773) = 1773, p<.001; Step 2: Adj R^2^ = .06, F(1, 1772) = 21.50, p<.001; Step 3: Adj R^2^ = .18, F(1, 1771) = 268.77, p<.001; Step 4: Adj R^2^ = .18, F(1, 1170) = 0.14, p=.71.

Supplementary notes for **Table 2**

*Wellbeing* Wave 4; Step 1: Adj R^2^ = .03; F(5, 2371) = 15.39, p<.001; Step 2: Adj R^2^ = .03, F(1, 2370) = 8.73, p=.003; Step 3: Adj R^2^ = .13, F(1, 2369) = 254.59, p<.001; Step 4: Adj R^2^ = .13, F(1, 2368) = 2.10, p=.15; Wave 6 Step 1: Adj R^2^ = .04; F(5, 2057) = 15.94, p<.001; Step 2: Adj R^2^ = .04, F(1, 2056) = 8.36, p=.004; Step 3: Adj R^2^ = .13, F(1, 2055) = 219.92, p<.001; Step 4: Adj R^2^ = .13, F(1, 2054) = 1.05, p=.31; Wave 8; Step 1: Adj R^2^ = .03; F(5, 1773) = 9.26, p<.001; Step 2: Adj R^2^ = .03, F(1, 1772) = 7.80, p=.005; Step 3: Adj R^2^ = .10, F(1, 1771) = 133.98, p<.001; Step 4: Adj R^2^ = .10, F(1, 1770) = 0.31, p=.58. *Loneliness* Wave 4; Step 1: Adj R^2^ = .04; F(5, 2371) = 19.71, p<.001; Step 2: Adj R^2^ = .04, F(1, 2370) = 1.92, p=.17; Step 3: Adj R^2^ = .10, F(1, 2369) = 149.84, p<.001; Step 4: Adj R^2^ = .10, F(1, 2368) = 0.42, p=.52; Wave 6 Step 1: Adj R^2^ = .04; F(5, 2057) = 18.50, p<.001; Step 2: Adj R^2^ = .04, F(1, 2056) = 5.03, p=.025; Step 3: Adj R^2^ = .10, F(1, 2055) = 129.26, p<.001; Step 4: Adj R^2^ = .10, F(1, 2054) = 0.05, p=.0.83; Wave 8; Step 1: Adj R^2^ = .03; F(1, 1773) = 11.59, p<.001; Step 2: Adj R^2^ = .03, F(1, 1772) = 10.15, p=.001; Step 3: Adj R^2^ = .09, F(1, 1771) = 106.65, p<.001; Step 4: Adj R^2^ = .09, F(1, 1770) = 0.04, p=.846.

Supplementary Table 3. Logistic regression analyses investigating the associations between probable COVID-19 infection during waves 1-3 (Mar-May 2020) and clinically meaningful cuff-offs for depression and anxiety at 1 month (May/June 2020), 5 month (Oct/Nov 2020) and 13 month (June/July 2021) follow up waves

|  | **Wave 4**  **May-June 2020** | | | | **Wave 6**  **Oct-Nov 2020** | | | | **Wave 8**  **June-July 2021** | | | |
| --- | --- | --- | --- | --- | --- | --- | --- | --- | --- | --- | --- | --- |
|  |  | *95% CI* | |  |  | *95% CI* | |  |  | *95% CI* | |  |
|  | **OR** | **Lower** | **Upper** | **p-value** | **OR** | **Lower** | **Upper** | **p-value** | **OR** | **Lower** | **Upper** | **p-value** |
| **Depression** |  |  |  |  |  |  |  |  |  |  |  |  |
| Age | 0.59 | 0.46 | 0.76 | <0.001 | 0.58 | 0.43 | 0.76 | <0.001 | 0.65 | 0.47 | 0.90 | 0.008 |
| SEG | 1.13 | 0.92 | 1.40 | 0.253 | 1.18 | 0.93 | 1.48 | 0.172 | 1.37 | 1.06 | 1.76 | 0.015 |
| Gender | 0.71 | 0.57 | 0.88 | 0.002 | 0.71 | 0.56 | 0.90 | 0.004 | 0.87 | 0.68 | 1.13 | 0.300 |
| Ethnicity | 1.29 | 0.88 | 1.90 | 0.188 | 1.40 | 0.90 | 2.16 | 0.137 | 2.32 | 1.48 | 3.62 | <0.001 |
| Physical health | 1.34 | 1.06 | 1.69 | 0.013 | 1.43 | 1.11 | 1.83 | 0.005 | 1.35 | 1.03 | 1.77 | 0.029 |
| COVID | 1.70 | 1.27 | 2.27 | <0.001 | 1.82 | 1.32 | 2.51 | <0.001 | 2.01 | 1.43 | 2.82 | <0.001 |
| Mental health | 4.64 | 3.74 | 5.75 | <0.001 | 5.04 | 3.98 | 6.37 | <0.001 | 4.24 | 3.27 | 5.48 | <0.001 |
| **Anxiety** |  |  |  |  |  |  |  |  |  |  |  |  |
| Age | 0.58 | 0.44 | 0.76 | <0.001 | 0.67 | 0.49 | 0.91 | 0.011 | 0.65 | 0.45 | 0.92 | 0.016 |
| SEG | 1.07 | 0.85 | 1.35 | 0.555 | 0.93 | 0.72 | 1.21 | 0.596 | 1.03 | 0.77 | 1.36 | 0.865 |
| Gender | 0.69 | 0.54 | 0.88 | 0.003 | 0.53 | 0.41 | 0.70 | <0.001 | 0.75 | 0.56 | 1.00 | 0.051 |
| Ethnicity | 0.97 | 0.62 | 1.50 | 0.882 | 1.00 | 0.59 | 1.69 | 0.997 | 1.27 | 0.73 | 2.22 | 0.398 |
| Physical health | 1.28 | 1.00 | 1.65 | 0.054 | 1.46 | 1.11 | 1.91 | 0.006 | 1.33 | 0.98 | 1.80 | 0.067 |
| COVID | 1.61 | 1.18 | 2.19 | 0.002 | 1.56 | 1.10 | 2.22 | 0.013 | 1.67 | 1.14 | 2.44 | 0.008 |
| Mental health | 4.28 | 3.39 | 5.39 | <0.001 | 4.75 | 3.67 | 6.13 | <0.001 | 4.87 | 3.67 | 6.47 | <0.001 |

Note: COVID status = yes (1) or no (0); gender = female (1), male (2); age group = less than or equal to 30 (1) or over 30 (2); ethnicity = White (1) or minority ethnic groups (2); socioeconomic group = high (1) or low (2); physical health conditions reported = no (0) or yes (1), mental health conditions reported: no (0) or yes (1). Step 3 results shown above. *Depression* Wave 4; Nagelkerke R Square = .18; X^2^ = 195.92, df= 1 p<.001; Wave 6; Nagelkerke R Square = .19; X^2^ = 184.11, df= 1 p<.001; Wave 8; Nagelkerke R Square = .16; X^2^ = 119.54, df= 1 p<.001. *Anxiety* Wave 4; Nagelkerke R Square = .16; X^2^ = 151.27, df= 1 p<.001; Wave 6; Nagelkerke R Square = .17; X^2^ = 142.71, df= 1 p<.001; Wave 8; Nagelkerke R Square = .15; X^2^ = 119.05, df= 1 p<.001.

Supplementary Table 4. Logistic regression analyses investigating the associations between probable COVID-19 infection during waves 1-3 (Mar-May 2020) and clinically meaningful cuff-offs for wellbeing and loneliness at 1 month (May/June 2020), 5 month (Oct/Nov 2020) and 13 month (June/July 2021) follow up waves

|  | **Wave 4**  **May-June 2020** | | | | **Wave 6**  **Oct-Nov 2020** | | | | **Wave 8**  **June-July 2021** | | | |
| --- | --- | --- | --- | --- | --- | --- | --- | --- | --- | --- | --- | --- |
|  |  | *95% CI* | |  |  | *95% CI* | |  |  | *95% CI* | |  |
|  | **OR** | **Lower** | **Upper** | **p-value** | **OR** | **Lower** | **Upper** | **p-value** | **OR** | **Lower** | **Upper** | **p-value** |
| **Wellbeing** |  |  |  |  |  |  |  |  |  |  |  |  |
| Age | 1.50 | 1.18 | 1.89 | <0.001 | 1.41 | 1.09 | 1.83 | 0.009 | 1.26 | 0.92 | 1.71 | 0.148 |
| SEG | 0.64 | 0.69 | 1.02 | 0.074 | 0.88 | 0.72 | 1.09 | 0.237 | 0.78 | 0.62 | 0.99 | 0.042 |
| Gender | 1.06 | 0.86 | 1.30 | 0.588 | 1.16 | 0.94 | 1.44 | 0.165 | 1.10 | 0.86 | 1.39 | 0.462 |
| Ethnicity | 0.72 | 0.50 | 1.01 | 0.060 | 0.65 | 0.44 | 0.95 | 0.026 | 0.50 | 0.33 | 0.76 | 0.001 |
| Physical health | 1.03 | 0.83 | 1.28 | 0.802 | 1.01 | 0.81 | 1.27 | 0.902 | 1.04 | 0.80 | 1.34 | 0.787 |
| COVID | 0.69 | 0.52 | 0.90 | 0.007 | 0.68 | 0.50 | 0.91 | 0.010 | 0.70 | 0.50 | 0.98 | 0.040 |
| Mental health | 0.28 | 0.23 | 0.23 | <0.001 | 0.30 | 0.24 | 0.38 | <0.001 | 0.29 | 0.23 | 0.37 | <0.001 |
| **Loneliness** |  |  |  |  |  |  |  |  |  |  |  |  |
| Age | 0.85 | 0.66 | 1.09 | 0.206 | 0.80 | 0.60 | 1.07 | 0.127 | 0.914 | 0.65 | 1.27 | 0.565 |
| SEG | 1.18 | 0.95 | 1.45 | 0.131 | 1.35 | 1.07 | 1.70 | 0.012 | 1.44 | 1.12 | 1.85 | 0.005 |
| Gender | 0.75 | 0.61 | 0.94 | 0.011 | 0.75 | 0.59 | 0.95 | 0.019 | 0.76 | 0.59 | 0.98 | 0.034 |
| Ethnicity | 1.10 | 0.75 | 1.61 | 0.642 | 1.20 | 0.77 | 1.87 | 0.430 | 1.40 | 0.86 | 2.27 | 0.171 |
| Physical health | 0.95 | 0.75 | 1.20 | 0.651 | 0.94 | 0.73 | 1.21 | 0.615 | 1.21 | 0.92 | 1.58 | 0.170 |
| COVID | 1.01 | 0.74 | 1.38 | 0.943 | 0.98 | 0.69 | 1.39 | 0.904 | 1.14 | 0.79 | 1.65 | 0.497 |
| Mental health | 2.94 | 2.36 | 3.65 | <0.001 | 3.26 | 2.57 | 4.14 | <0.001 | 3.03 | 2.34 | 3.93 | <0.001 |

Note: COVID status = yes (1) or no (0); gender = female (1), male (2); age group = less than or equal to 30 (1) or over 30 (2); ethnicity = White (1) or minority ethnic groups (2); socioeconomic group = high (1) or low (2); physical health conditions reported = no (0) or yes (1), mental health conditions reported: no (0) or yes (1). Step 3 results shown above. *Wellbeing* Wave 4; Nagelkerke R Square = .12; X^2^ = 148.25, df= 1 p<.001; Wave 6; Nagelkerke R Square = .11; X^2^ = 113.08, df= 1 p<.001; Wave 8; Nagelkerke R Square = .11; X^2^ = 95.09, df= 1 p<.001. *Loneliness* Wave 4; Nagelkerke R Square = .08; X^2^ = 92.57, df= 1 p<.001; Wave 6; Nagelkerke R Square = .10; X^2^ = 92.25, df= 1 p<.001; Wave 8; Nagelkerke R Square = .09; X^2^ = 67.57, df= 1 p<.001.
